# A dynamic modelling analysis of the impact of tobacco control programs on population-level nicotine dependence

**DOI:** 10.1038/s41598-021-81460-9

**Published:** 2021-01-21

**Authors:** Adam Skinner, Jo-An Occhipinti, Nathaniel D. Osgood

**Affiliations:** 1 Brain and Mind Centre, Faculty of Medicine and Health, University of Sydney, Sydney, Australia; 2 Department of Computer Science, University of Saskatchewan, Saskatoon, Canada

**Keywords:** Risk factors, Mathematics and computing

## Abstract

According to the ‘hardening hypothesis’, average nicotine dependence will increase as less dependent smokers quit relatively easily in response to effective public health interventions, so that sustained progress in reducing smoking prevalence will depend on shifting the emphasis of tobacco control programs towards intensive treatment of heavily dependent smokers (who comprise an increasing fraction of continuing smokers). We used a system dynamics model of smoking behaviour to explore the potential for hardening in a population of smokers exposed to effective tobacco control measures over an extended period. Policy-induced increases in the per capita cessation rate are shown to lead inevitably to a decline in the proportion of smokers who are heavily dependent, contrary to the hardening hypothesis. Changes in smoking behaviour in Australia over the period 2001‒2016 resulted in substantial decreases in current smoking prevalence (from 23.1% in 2001 to 14.6% in 2016) and the proportion of heavily dependent smokers in the smoking population (from 52.1% to 36.9%). Public health interventions that have proved particularly effective in reducing smoking prevalence (tobacco tax increases, smoke-free environment legislation, antismoking mass media campaigns) are expected to also contribute to a decline in population-level nicotine dependence.

## Introduction

Nearly 6.5 million people are estimated to have died from smoking-related diseases in 2015, corresponding to 11.5% of global mortality that year^1^. Despite this appalling statistic, substantial progress has been made in reducing smoking prevalence over the past three decades. Particularly dramatic declines in smoking prevalence have been achieved in countries where effective public health measures (e.g., taxation, mass media campaigns, smoke-free legislation) have been in place for an extended period, leading some researchers to propose that the smoking populations of these countries may become increasingly less responsive to such measures as prevalence continues to decline, since many smokers who could be prompted to quit will have already done so^2–4^. According to this ‘hardening hypothesis’, sustained progress in reducing smoking prevalence will depend upon shifting the emphasis of tobacco control programs towards intensive treatment of individual smokers^3^.

Empirical evidence for the hardening hypothesis comes primarily from analyses showing an inverse association between population smoking prevalence and nicotine dependence across (mostly European) countries, and studies indicating a decline in the effectiveness of smoking cessation treatments in clinical trials^2,4‒6^. These results point to a decrease in the ability of smokers to quit as smoking prevalence declines, consistent with hardening. However, recent analyses of population survey data from the United States, Europe, and New Zealand provide no evidence that significant declines in smoking prevalence in these regions have been associated with declines in smokers’ motivation or capacity to quit^7–9^. Proportions of New Zealand daily smokers who had not attempted to quit in the past year or had made multiple unsuccessful cessation attempts (an indicator of high nicotine dependence) did not change significantly between 2008 and 2014, despite a decrease in smoking prevalence from 16.9% to 14.4% over this period^8^. In the United States, both the proportion of daily smokers who had not made a cessation attempt in the past year and the mean number of cigarettes smoked per day decreased as smoking prevalence declined from 19.6% in 1993 to 12.7% in 2011^7^.

Although it may appear self-evident that population-level nicotine dependence should increase as less dependent smokers quit at a higher rate than heavily dependent smokers (who comprise an increasing proportion of continuing smokers), this argument, which underpins the hardening hypothesis, effectively ignores the potential for initiation and changes in smoking intensity (i.e., cigarette consumption) to alter the distribution of nicotine dependence in a smoking population^10,11^. Population-level nicotine dependence may increase or decrease as smoking prevalence declines in response to effective tobacco control interventions, depending on the combined (potentially opposing) effects of the underlying dynamics of initiation, smoking intensity, cessation, relapse, and smoking-related mortality. This paper presents an analysis of the conditions under which we would anticipate hardening of a population of smokers exposed to a comprehensive tobacco control program over an extended period. Using a simple system dynamics model of smoking behaviour, we demonstrate via a series of simulation experiments and a detailed case study that hardening (defined here as an increase in the proportion of smokers who are heavily dependent) would be expected only under a restricted set of conditions that will rarely apply in mature tobacco control policy environments.

Methods

### Model structure and assumptions

**Figure 1:**
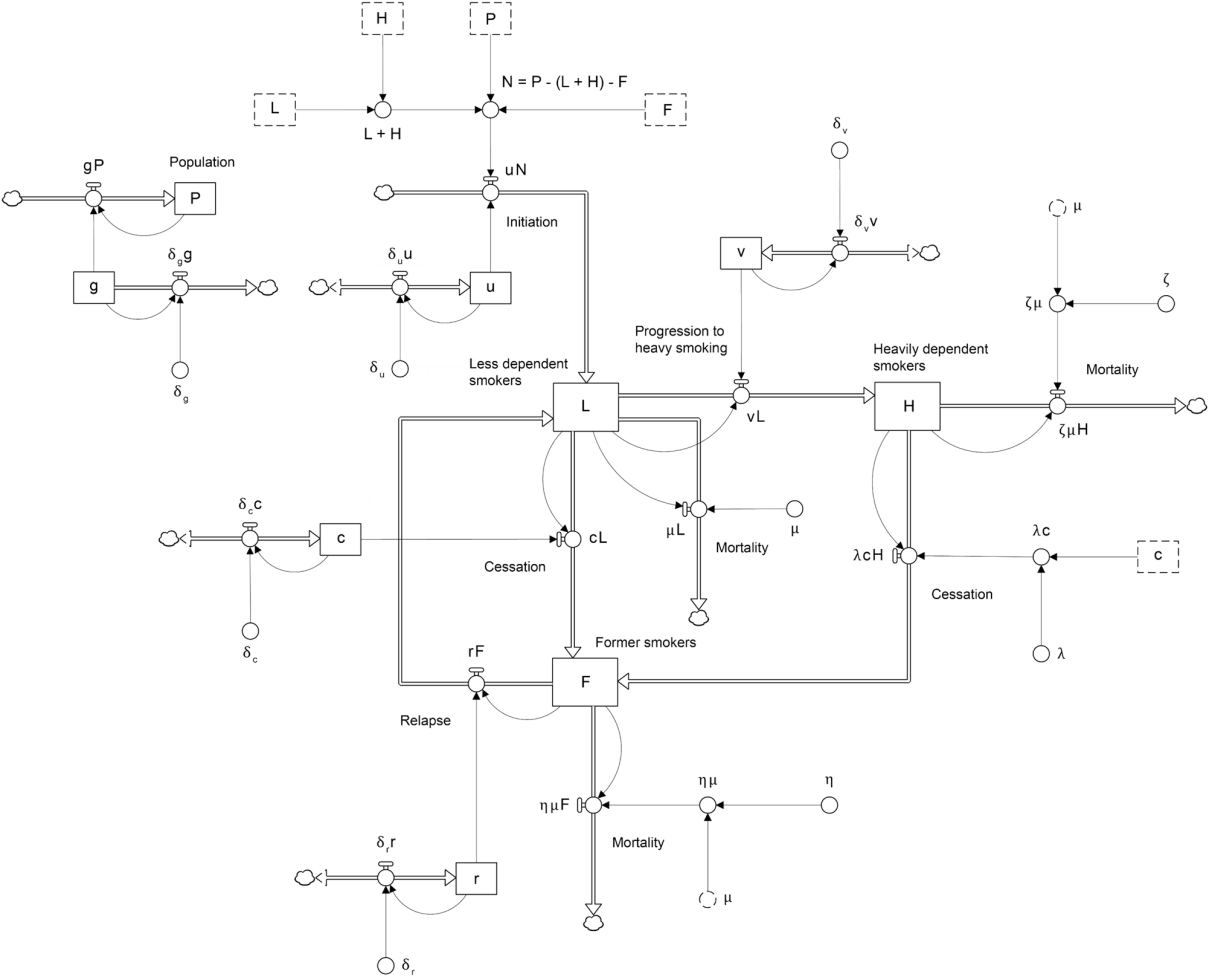
System dynamics model of smoking behaviour used for the simulation experiments and case study. Symbols are defined in the Methods section and Table 1.

Figure 1 presents the system dynamics model used for the simulation experiments and case study described below. The core of the model comprises three stocks (i.e., state variables), corresponding to the numbers of less dependent smokers ($L$), heavily dependent smokers ($H$), and former smokers ($F$) in the total population ($P$). Newly recruited smokers are added to the stock of less dependent smokers at a rate (per year) equal to the number of never smokers ($N = P - L - H - F$) multiplied by the per capita initiation rate $u$. Less dependent smokers progress to heavily dependent smoking at a per capita rate $v$, so that $vL$ less dependent smokers become heavily dependent smokers per year. As smokers stop smoking, they flow into the stock of former smokers; cessation rates for less dependent and heavily dependent smokers are equal to $cL$ and $\lambda cH$, respectively, where $c$ is the per capita cessation rate for less dependent smokers, and the cessation rate ratio $\lambda$ is assumed to be less than 1. Relapsing former smokers are assumed to return to the stock of less dependent smokers at a per capita rate $r$, irrespective of their level of dependence prior to quitting. This assumption implies that former smokers have remained abstinent for a sufficient period that their past smoking behaviour no longer predicts their risk of relapse (*c*. 1 month or more)^12^.

Per capita mortality rates for less dependent, heavily dependent, and former smokers are equal to $\mu$, $\zeta \mu$, and $\eta \mu$, respectively, where we assume $\zeta$ is greater than 1 and $\eta$ is less than 1 (i.e., the per capita mortality rate for less dependent smokers is assumed to be lower than that for heavily dependent smokers and greater than the rate for former smokers). Never smokers are added to the total population ($P$) at a rate equal to $gP$ per year; for simplicity, changes in the numbers of less dependent, heavily dependent, and former smokers due to migration are assumed to be negligible. The fractional population growth rate $g$ was set to zero for the simulation experiments presented here (i.e., we assumed a constant total population; see Table 1); for the case study, $g$ was assumed to decrease by a constant fraction $\delta_{g}$ per year to accommodate declining population growth over time (see Supplementary appendix 1).

**Table 1:** Parameter values assumed in the simulation experiments.

Parameter	Symbol	Value
Initial population	*P*_0_	1,000,000
Initial fractional population growth rate	*g*_0_	0
Fractional rate of decrease in the population growth rate	*δ*_*g*_	0
Initial number of less dependent smokers	*L*_0_	40,000
Initial number of heavily dependent smokers	*H*_0_	60,000
Initial number of former smokers	*F*_0_	150,000
Per capita mortality rate for less dependent smokers	*μ*	0.02
Mortality rate ratio for heavily dependent smokers	*ζ*	1.5
Mortality rate ratio for former smokers	*η*	0.5
Cessation rate ratio for heavily dependent smokers	*λ*	0.4
Initial per capita initiation rate	*u*_0_	0.01
Initial per capita rate of progression to heavily dependent smoking	*v*_0_	0.2
Initial per capita cessation rate for less dependent smokers	*c*_0_	0.4
Initial per capita relapse rate	*r*_0_	0.2
Fractional rate of increase in the per capita initiation rate	*δ*_*u*_	0
Fractional rate of increase in the per capita rate of progression to heavily dependent smoking	*δ*_*v*_	0
Fractional rate of increase in the per capita cessation rate for less dependent smokers	*δ*_*c*_	0
Fractional rate of increase in the per capita relapse rate	*δ*_*r*_	0

### Simulation experiments

The potential for hardening accompanying policy-induced changes in rates of initiation, progression to heavily dependent smoking, cessation, and relapse was explored via a series of simulation experiments. Each experiment involved running the system dynamics model for a sufficient period that the proportion of heavily dependent smokers in the total smoking population, $q = H/\left( {L + H} \right)$, reached equilibrium (10^3^ model years in the simulations reported here), then increasing or decreasing one of the per capita rates $u$, $v$, $c$, or $r$ (defined in the previous section), and observing the effect on $q$ over time. All rate changes were instantaneous and sustained; i.e., the focal per capita rate was effectively modelled as a step function of time. Note that changes to the per capita cessation rate for less dependent smokers, $c$, alter the total cessation rate without affecting the ratio of the per capita cessation rates for less dependent and heavily dependent smokers (which are equal to $c$ and $\lambda c$, respectively). Table 1 shows the parameter values assumed for the simulation experiments reported here. Results qualitatively similar to those presented below are obtained for a wide range of parameter values, so our general conclusions are not dependent upon the particular assumptions in Table 1 (see Supplementary appendix 2).

### Case study: smoking behaviours in Australia

The simulation experiments described above provide a means of examining the effects of independent changes in rates of initiation, progression to heavily dependent smoking, cessation, and relapse on the proportion of heavily dependent smokers in a smoking population; however, the potential for hardening in real populations of smokers will generally depend on the combined effects of concurrent changes in most or all of these rates. To examine the impacts of a multifaceted tobacco control program on rates of smoking behaviour change and their combined effect on the proportion of smokers who are heavily dependent, we used Bayesian Markov chain Monte Carlo (MCMC) simulation^13^ to fit the system dynamics model in Fig. 1 to National Drug Strategy Household Survey (NDSHS) data on smoking behaviours in Australia over the period 2001‒2016^14^.

Australian governments have adopted a broad range of tobacco control policies over the past 40 years, including tobacco advertising and sponsorship bans, smoke-free environment legislation, taxation on tobacco products, and significant investment in antismoking mass media campaigns and cessation support services^15^. The effects of these polices on smoking behaviours were modelled as fractional increases (potentially negative) in per capita rates of initiation, progression to heavily dependent smoking, cessation, and relapse per year; thus, for example, the rate of increase in the per capita cessation rate for less dependent smokers, $c$, is equal to $\delta_{c} c$, where $\delta_{c}$ is the fractional increase in $c$ per year (the corresponding rates of increase in the per capita rates of initiation, progression to heavily dependent smoking, and relapse are $\delta_{u} u$, $\delta_{v} v$, and $\delta_{r} r$, respectively) (see Fig. 1). Note that the per capita rates $u$, $v$, $c$, and $r$ will decrease over time where their fractional rate of increase is negative.

Time series data on national numbers of current and former smokers aged 14 years and above, numbers of pack-a-day smokers (i.e., those smoking 20 or more cigarettes per day), and numbers of current and former smokers who quit smoking for one month or more in the past year were used to estimate the fractional increase rates $\delta_{u}$, $\delta_{v}$, $\delta_{c}$, and $\delta_{r}$, initial values for the per capita rates $u$, $v$, $c$, and $r$ (i.e., the rates applying at the start of 2001), initial numbers of current and former smokers, and the initial proportion of smokers who are heavily dependent (i.e., the value of $q$ at the start of 2001). Letting $y_{i} \left( t \right)$ and $m_{i} \left( {t, \theta } \right)$ denote, respectively, the observed value of time series $i$ at time $t$ (e.g., the number of pack-a-day smokers from the NDSHS data for 2010) and the corresponding simulation model output (the modelled number of heavily dependent smokers in 2010) obtained for a particular set of parameter values $\theta$, the likelihood for each $y_{i} \left( t \right)$ was calculated as $p\left( {y_{i} \left( t \right)|\theta , \varepsilon_{i} \left( t \right), \sigma_{i} } \right) = Poisson\left( {y_{i} \left( t \right)|m_{i} \left( {t, \theta } \right)e^{{\varepsilon_{i} \left( t \right)}} } \right)$, where $\varepsilon_{i} \left( t \right) \sim N\left( {0, \sigma_{i}^{2} } \right)$; i.e., we assumed that the data $y_{i} \left( t \right)$ follow an overdispersed Poisson distribution. Deviations of the observed data from their expected value were assumed to be independent, so the likelihood function for the combined time series data is the product of the likelihoods for all $y_{i} \left( t \right)$. Posterior simulation was performed using Stan ver. 2.19.2 (see Supplementary appendix 3)^16^.

## Results

### Simulation experiments

**Figure 2:**
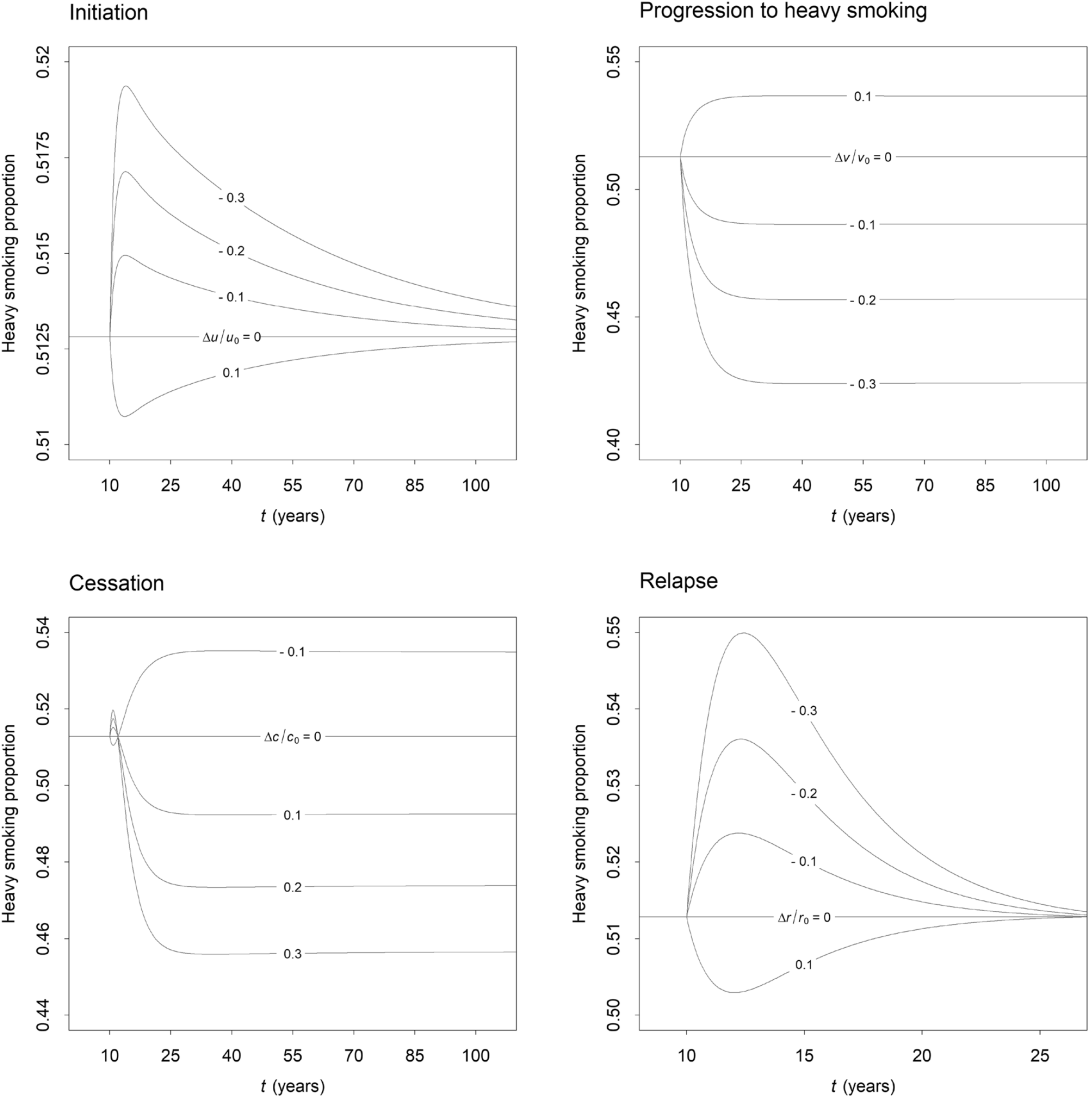
Effects of changes in per capita rates of smoking behaviour change on the proportion of smokers who are heavily dependent. All rate changes were instantaneous and sustained (starting at $t = 10$). Per capita rates of initiation, progression to heavily dependent smoking, and relapse ($u$, $v$, and $r$, respectively) were reduced by 10%, 20%, and 30% of their initial values ($u_{0}$, $v_{0}$, and $r_{0}$), and increased by 10% of their initial values; the per capita cessation rate for less dependent smokers, $c$, was increased by 10%, 20%, and 30% of its initial value ($c_{0}$) and reduced by 10% of its initial value.

Figure 2 shows the effects of sustained changes in rates of smoking behaviour change on $q$ (i.e., the proportion of heavily dependent smokers in the current smoking population) observed in the simulation experiments. Assuming the per capita initiation rate is positive, the equilibrium value of $q$ is equal to $v/\left( {v + \lambda c + \zeta \mu } \right)$, so increases in the per capita cessation rate for less dependent smokers, $c$, lead to a decrease in the equilibrium proportion of smokers who are heavily dependent (i.e., we expect softening of the smoking population, not hardening; see Supplementary appendix 4). Nevertheless, Fig. 2 shows that when $c$ is increased, $q$ also increases initially, before declining to the new equilibrium value (note, however, that the total decrease in $q$ is generally substantially greater than the initial increase). Reductions in the per capita rate of progression to heavily dependent smoking, $v$, also reduce the equilibrium value of $q$ (since the proportional decrease in the denominator, equal to $- \Delta v/\left( {v + \lambda c + \zeta \mu } \right)$, is less than the proportional decrease in the numerator, i.e., $- \Delta v/v$), although in this case, the proportion of heavily dependent smokers in the total smoking population declines consistently to the new equilibrium proportion.

Decreases in the per capita initiation and relapse rates ($u$ and $r$, respectively) produce an immediate increase in the proportion of smokers who are heavily dependent; however, $q$ eventually reaches a maximum and then declines towards its initial value (51.3% of smokers). Both initiation and relapse add to the stock of less dependent smokers, so the rate of increase in the number of less dependent smokers decreases when either the per capita initiation rate or the per capita relapse rate is reduced, prompting an increase in $q$. The (stable) equilibrium value of $q$ does not depend on the per capita initiation and relapse rates, however, so after initially increasing, the proportion of smokers who are heavily dependent declines to its initial value as the system returns to equilibrium. Larger reductions in the per capita initiation and relapse rates generally produce greater temporary increases in $q$, while temporary decreases in $q$ are observed when $u$ and $r$ are increased (Fig. 2; qualitatively similar results are observed for changes in per capita rates of cessation and progression to heavily dependent smoking).

### Case study

**Figure 3:**
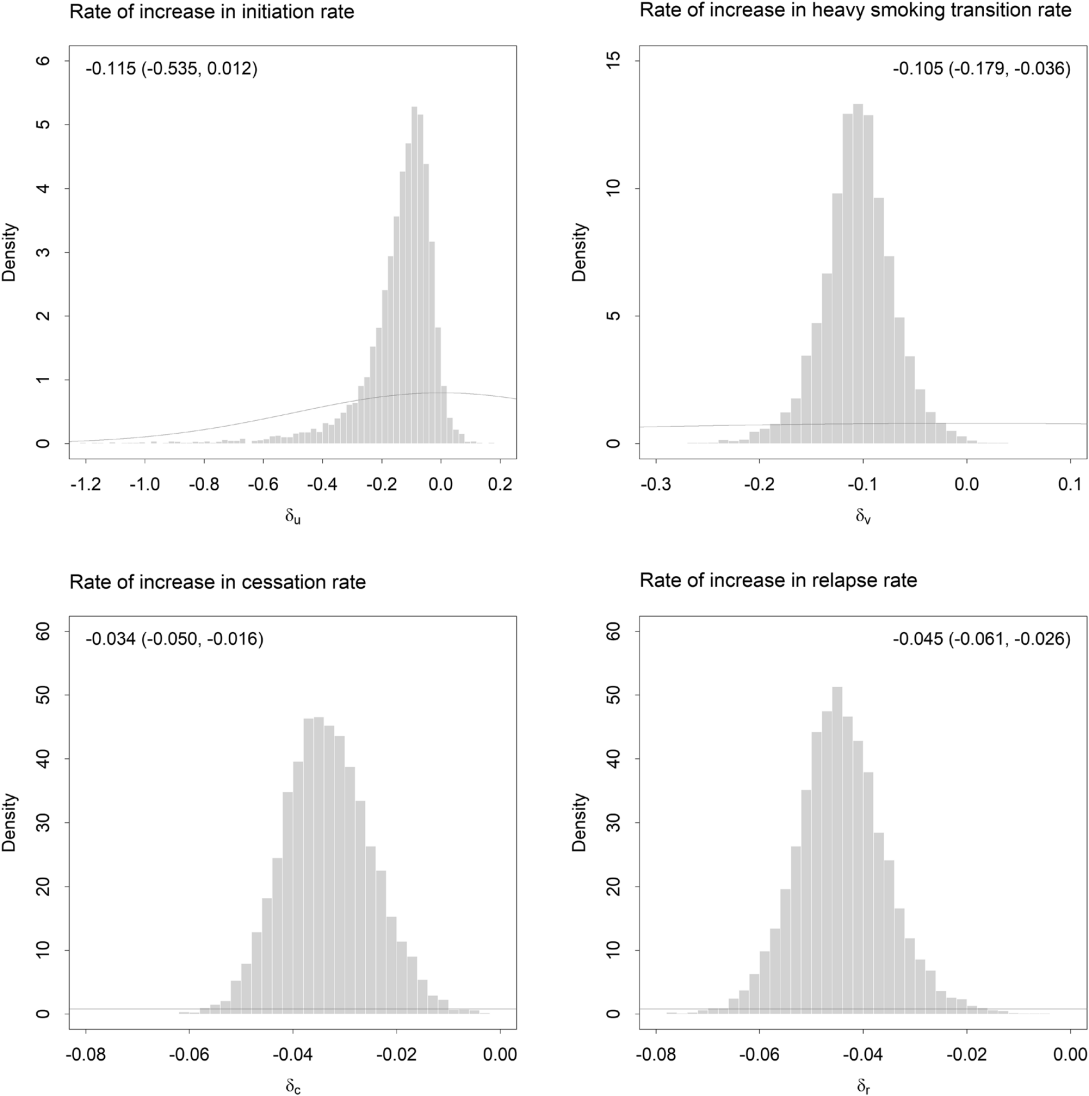
Marginal posterior distributions inferred for fractional rates of increase in per capita rates of smoking behaviour change in the Australian smoking population over the period 2001‒2016. The histograms were generated from 8,000 parameter vectors ($\theta$) sampled from the posterior distribution in the Markov chain Monte Carlo (MCMC) analysis. Median estimates and 95% posterior intervals are shown in the top left or right corner. Prior distributions are plotted as smooth curves.

**Figure 4:**
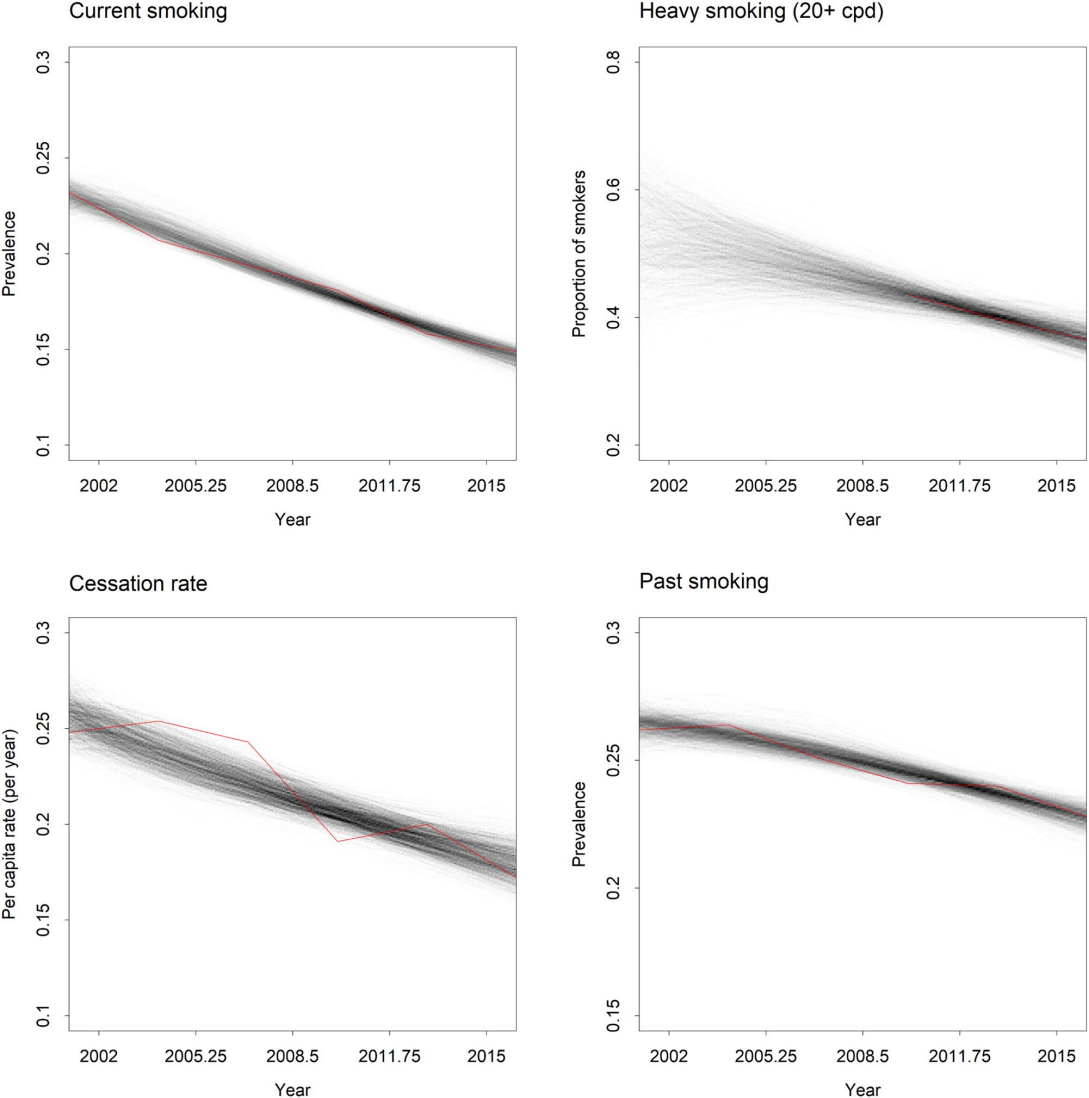
Time-specific posterior density estimates for current smoking prevalence, the proportion of smokers who are heavily dependent, the per capita cessation rate per year, and past smoking prevalence in the Australian population (aged 14 years and above) over the period 2001‒2016. Density estimates were derived from 10^3^ simulations, each of which used a randomly chosen parameter vector ($\theta$) sampled in the Markov chain Monte Carlo (MCMC) analysis. Time series data from the National Drug Strategy Household Survey (NHSHS) are plotted in red (note that data on the proportion of smokers who are heavily dependent were available for 2010‒2016 only).

Posterior distributions for the fractional increase rates $\delta_{u}$, $\delta_{v}$, $\delta_{c}$, and $\delta_{r}$ derived from the MCMC analysis are presented in Fig. 3 (posterior distributions for the remaining simulation model parameters are presented in Supplementary appendix 5). Median estimates for all fractional increase rates are negative, indicating that per capita rates of initiation, progression to heavily dependent smoking, cessation, and relapse declined over the period 2001‒2016; 95% posterior intervals for all rates except $\delta_{u}$, i.e., the fractional rate of increase in the per capita initiation rate, exclude 0 (Fig. 3). Figure 4 presents time-specific posterior density estimates for selected model outputs (current and past smoking prevalence, the proportion of heavy smokers in the smoking population, and the per capita cessation rate per year) derived from 10^3^ simulations, each of which used a randomly chosen parameter vector $\theta$ sampled in the MCMC analysis. The proportion of heavily dependent smokers in the smoking population is estimated only imprecisely over the early part of the simulation period (since NDSHS data on numbers of pack-a-day smokers were only available for 2010‒2016) but declines consistently from *c*. 2006 onwards. Current smoking prevalence, past smoking prevalence, and the per capita cessation rate (i.e., the number of successful quit attempts per smoker per year) decline more or less steadily from 2001 to 2016.

## Discussion

The simulation experiments and case study results presented above provide support for a number of conclusions about the potential for hardening in a population of smokers exposed to a comprehensive tobacco control program over an extended period. First, the principal argument underpinning the hardening hypothesis, i.e., that the proportion of heavily dependent smokers in a smoking population will increase as less dependent smokers quit more rapidly in response to effective public health policies, does not hold generally. Although an increase in cessation may prompt an initial increase in the proportion of smokers who are heavily dependent, $q$, the equilibrium value of $q$ decreases as the per capita cessation rate $c$ increases, so any persistent intervention that promotes cessation would be expected to ultimately result in softening of a population of smokers, not hardening (Fig. 2; Supplementary appendix 4). Second, the near-term effects of changes in rates of initiation, progression to heavily dependent smoking, cessation, and relapse on the proportion of smokers who are heavily dependent may be antagonistic; e.g., $q$ increases initially when initiation and relapse rates are reduced, but decreases in response to a reduction in the rate of progression to heavily dependent smoking. The potential for hardening will therefore depend on the specific measures implemented as part of a tobacco control program, at least in the short term (ultimately, $q$ is expected to decline, however). And third, the ability of smokers to quit may decrease as the proportion of heavily dependent smokers in a smoking population declines, indicating that ‘hardening’ may occur through more than one mechanism.

Our case study results suggest that the comprehensive set of tobacco control measures adopted in Australia over the past *c*. 40 years, including all measures prescribed under the World Health Organisation’s Framework Convention on Tobacco Control (WHO FCTC)^17,18^, has contributed to declines in per capita rates of initiation, progression to heavily dependent smoking, and relapse, although per capita cessation rates among less dependent and heavily dependent smokers also decreased over the period 2001‒2016 (see Fig. 3). The combined effect of these changes in rates of smoking behaviour change has been a significant decline in both current smoking prevalence (from 23.1% in 2001 to 14.6% in 2016, based on mean estimates from the MCMC analysis) and the proportion of heavily dependent smokers in the smoking population (from 52.1% in 2001 to 36.9% in 2016). These results are largely consistent with the two recent analyses of smoking behaviour trends in the United States, Europe, and New Zealand described in the Introduction^7,8^, and suggest that the potential for hardening in smoking populations subjected to a range of WHO FCTC-prescribed tobacco control measures is minimal. Policy interventions that are effective in limiting cigarette consumption and promoting cessation (e.g., tobacco tax increases, smoke-free environment policies, antismoking mass media campaigns)^19–21^ in particular are expected to lead to a decline in the proportion of current smokers who are heavily dependent, while also reducing smoking prevalence (see Fig. 2).

The decline in the total per capita cessation rate among Australian smokers evident from Fig. 4 suggests that the average ability or motivation of smokers to quit is at least partially independent of changes in the proportion of smokers who are heavily dependent. Although the reasons for this decline are unclear, one possible contributing factor is a decrease in perceived self-efficacy attributable to the shifting demographic composition of the Australian smoking population, which has become increasingly dominated by socioeconomically disadvantaged smokers^22^. Perceived self-efficacy is a reliable predictor of success in quitting^23^, and is inversely associated with individual-level measures of socioeconomic disadvantage^24^, so that an increase in the proportion of disadvantaged smokers in a smoking population may be expected to result in a decrease in the per capita cessation rate. While the inferred decline in the total per capita cessation rate could be considered as a form of ‘hardening’^11^, it should be emphasised that the mechanism underlying this decline (i.e., a general decrease in the per capita cessation rate affecting both less dependent and heavily dependent smokers) differs from that proposed under the hardening hypothesis (an increase in the proportion of smokers who are heavily dependent due to a higher cessation rate among less dependent smokers).

### Limitations

Several potentially significant determinants of smoking behaviour in real populations, including social network effects^25^, the effect of smoking-related disease incidence on the cessation rate^26^, and demographic trends altering the composition of the smoking population over time were not modelled explicitly in the analyses presented here. Additionally, our approach to modelling the impacts of tobacco control polices on rates of smoking behaviour change in the Australian smoking population (i.e., as exponential growth or decay in per capita rates of initiation, progression to heavy smoking, cessation, and relapse) effectively disregards the potential for time-varying intervention effects and complex policy interactions over the simulation period. The substantial simplifying assumptions we have made in modelling the dynamics of smoking behaviour serve to ensure analytical tractability; however, they clearly do so at the expense of model realism. Analyses using more complex dynamic models^27,28^ are needed to assess the degree to which our conclusions about the potential for hardening in smoking populations depend on these assumptions. Nevertheless, it should be noted that our system dynamics model at least generates outputs corresponding closely with historical data from the NDSHS, despite its simplicity (see Fig. 4).

Population data on nicotine dependence were not available from the NDSHS, so we used estimates of numbers of pack-a-day smokers to infer the proportion of heavily dependent smokers in the Australian smoking population. Proponents of the hardening hypothesis have criticised the use of consumption-based measures as a proxy for nicotine dependence, arguing that many smokers will respond to a reduction in the number of cigarettes smoked per day by modifying their smoking behaviour, increasing the amount of nicotine inhaled per cigarette through compensatory smoking (i.e., drawing more frequently and deeply on each cigarette)^3^. An analysis of changes in daily cigarette consumption and serum cotinine concentrations in the United States smoking population between 1988 and 2012 provides evidence for decoupling of the mean number of cigarettes smoked per day and population-level nicotine dependence, consistent with this argument^29^. Nevertheless, individual-level analyses indicate that daily cigarette consumption is associated with biochemical markers of nicotine dependence (exhaled carbon monoxide and salivary cotinine)^30^ and is a significant predictor of relapse during the initial *c*. 1 month of a cessation attempt^12^. Pack-a-day smokers may therefore be expected to have relatively high levels of nicotine dependence and to quit at a lower rate than smokers smoking fewer cigarettes per day (i.e., the necessary conditions for hardening to occur via the mechanism proposed under the hardening hypothesis should be satisfied).

## Conclusion

The results of our simulation experiments and case study indicate that the potential for hardening in populations of smokers exposed to a comprehensive tobacco control program over an extended period is limited. Despite its intuitive appeal, the hardening hypothesis fails to take into account the indirect effects of an increase in the per capita cessation rate (e.g., a decrease in the rate of progression from less dependent to heavily dependent smoking) and does not hold generally. Public health policy measures that are effective in promoting cessation and limiting cigarette consumption are ultimately expected to contribute to a decrease in the proportion of smokers who are heavily dependent, as our simulation experiments demonstrate (Fig. 2). This conclusion is consistent with recent analyses of smoking behaviour trends in the United States and Europe^7,9^ and the Australian case study presented here, both of which provide evidence for concurrent declines in smoking prevalence and the proportion of smokers who are heavily dependent. Although we may reasonably assume that continuing success in reducing smoking-related harms will depend on the development of new tobacco control approaches and ongoing refinement of those measures already in place, our analyses provide no evidence to support a shift in the focus of tobacco control strategies towards intensive treatment of individual smokers. Rather, we conclude that established public health interventions that have proved effective in reducing smoking prevalence (tobacco tax increases, smoke-free environment legislation, mass media campaigns)^18^ will also contribute to a continuing decline in population-level nicotine dependence.

## Supplementary Information

Supplementary Information 1.
