# Supplementary material for: A dynamic modelling analysis of the impact of tobacco control programs on population-level nicotine dependence
Source: Sci Rep. 2021 Jan 21;11:1866. doi: 10.1038/s41598-021-81460-9 (PMC7820504; doi:10.1038/s41598-021-81460-9)
Supplement: Supplementary file 1 — Supplementary Information 1. [file 41598_2021_81460_MOESM1_ESM.docx]

Supplementary information. A dynamic modelling analysis of the impact of tobacco control programs on population-level nicotine dependence.

Adam Skinner^1,*^, Jo-An Occhipinti^1^, Nathaniel D. Osgood^2^

^1^ Brain and Mind Centre, Faculty of Medicine and Health, University of Sydney, Sydney, Australia; ^2^ Department of Computer Science, University of Saskatchewan, Saskatoon, Canada

^*^ Corresponding author. Email: adam.skinner@sydney.edu.au. Phone: +61 4 5045 8201.

Supplementary appendix 1

Estimation of population parameters for the case study

The initial fractional growth rate, $g_{0}$, for the total Australian population aged 14 years or more and the fractional rate of decline in $g$, $\delta_{g}$, were estimated via constrained optimisation, using population data published by the Australian Bureau of Statistics (Australian Bureau of Statistics, 2020). Values of $g$ and $\delta_{g}$ minimising the mean of the absolute differences between the published data values and the corresponding model outputs (i.e., the mean absolute error; see, e.g., Sterman, 2000) were obtained using Powell’s method (Powell, 2009). Population estimates derived from the simulation model are plotted alongside the Australian Bureau of Statistics estimates in Figure S1.


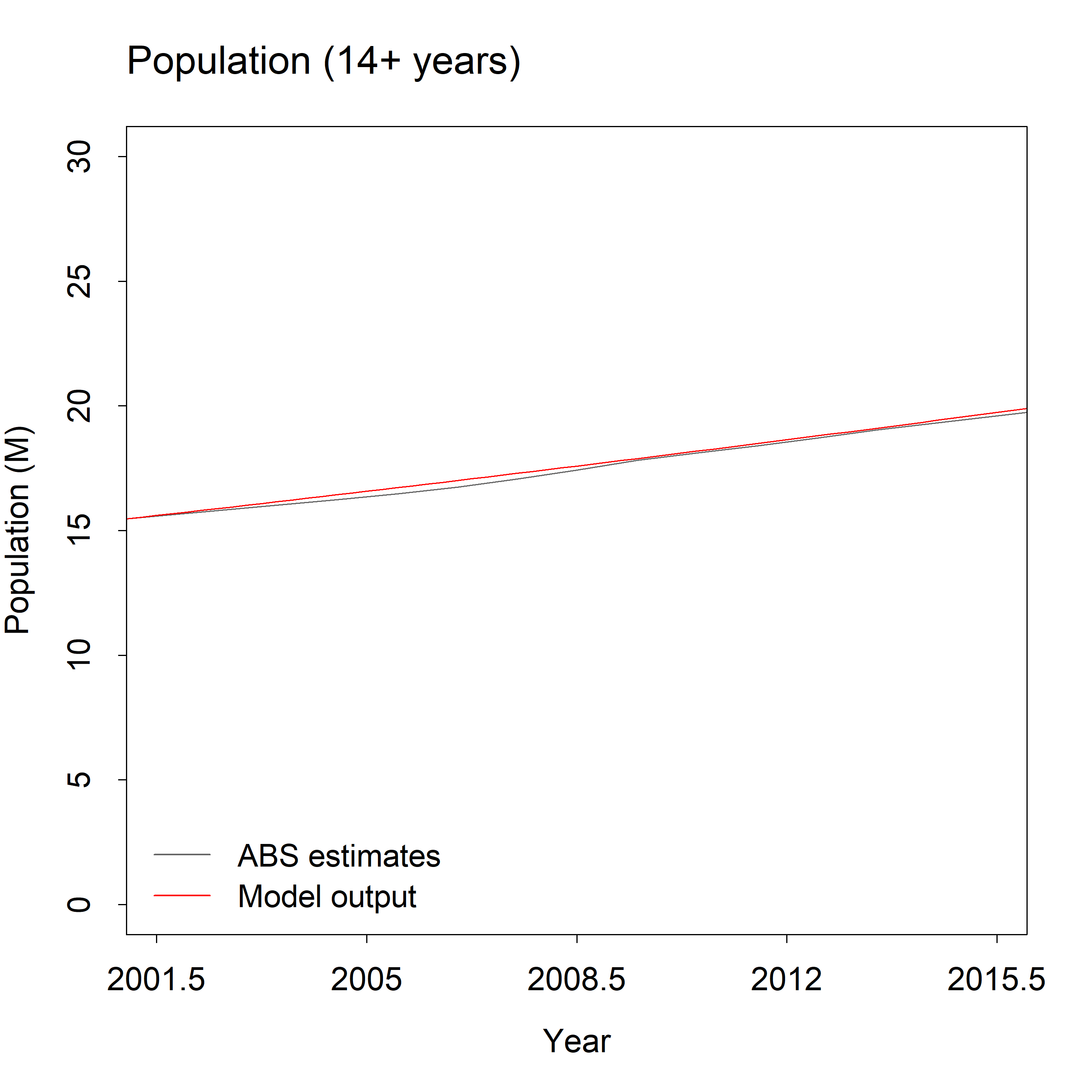


Figure S1. Population estimates derived from the system dynamics model and corresponding Australian Bureau of Statistics (ABS) estimates.

References

Australian Bureau of Statistics, 2020. Australian demographic statistics, Sep 2019. Cat. no. 3101.0. Australian Bureau of Statistics, Canberra.

Powell, M. J. D., 2009. The BOBYQA algorithm for bound constrained optimization without derivatives. Technical repot no. DAMTP 2009/NA06. Department of Applied Mathematics and Theoretical Physics, Cambridge University, Cambridge.

Sterman, J. D., 2000. Business dynamics: systems thinking and modeling for a complex world. McGraw-Hill, Boston.

Supplementary appendix 2

Sensitivity analyses for the simulation experiments

The extent to which the simulation results presented in Figure 2 of the paper depend on the assumed parameter values (see Table 1) was assessed via sensitivity analysis. Latin hypercube sampling was used to generate 100 sets of values for the initial per capita rates of smoking behaviour change ($u_{0}$, $v_{0}$, $c_{0}$, and $r_{0}$) from a relatively broad distribution of values (see Figure S2). For each set of initial values, we ran a series of simulation experiments in which the per capita rates $u$, $v$, $c$, and $r$ were separately decreased ($u$, $v$, and $r$) or increased ($c$) by 10% of their initial values (after running the model for a sufficient period that the proportion of heavily dependent smokers in the smoking population, $q$, reached equilibrium; see the Methods section of the paper). Figure S3 plots the difference over time between the proportion of smokers who are heavily dependent and the equilibrium proportion obtaining prior to the rate change ($q_{0}^{*}$) for each set of initial values (we plotted $q-q_{0}^{*}$ rather than $q$ as $q_{0}^{*}$, and hence the value of $q$ prior to the per capita rate shift, depends on $v_{0}$ and $c_{0}$; see Supplementary appendix 4). Despite the broad range of initial per capita rates, the effects of changes in $u$, $v$, $c$, and $r$ on $q$ are qualitatively similar across simulations (e.g., $q$ always increases briefly before declining rapidly when the per capita cessation rate $c$ is increased); the general conclusions derived from the simulation experiments presented in the paper are therefore not dependent upon the particular parameter values in Table 1. (Note that varying the initial numbers of less dependent, heavily dependent, and former smokers has no effect on the simulation results, as these stocks settle at their equilibrium values prior to the changes in $u$, $v$, $c$, and $r$.)


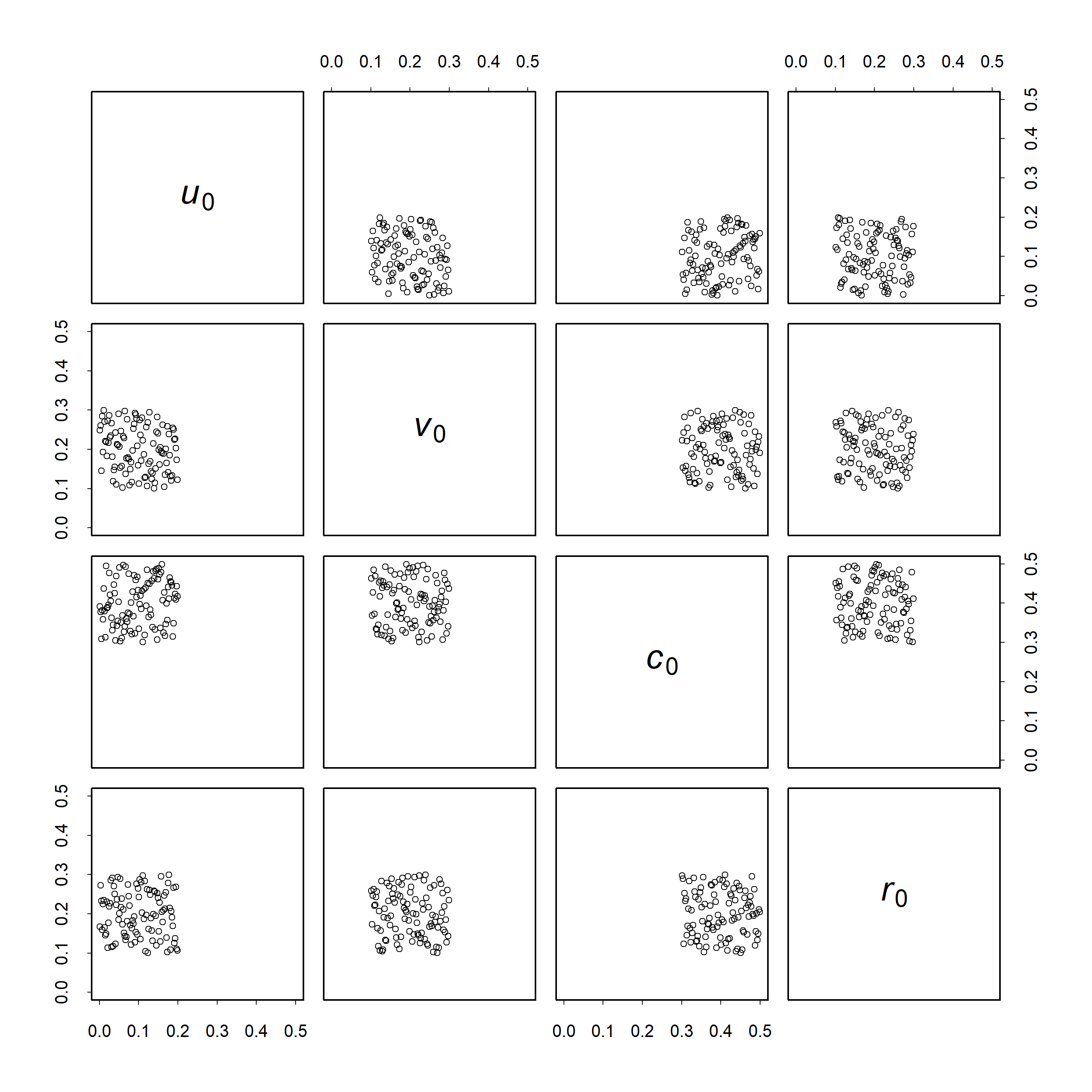


Figure S2. Scatterplot matrix showing values for the initial per capita rates of initiation, progression to heavily dependent smoking, cessation, and relapse ($u_{0}$, $v_{0}$, $c_{0}$, and $r_{0}$, respectively) used in the sensitivity analyses. Values were sampled from $\text{Uniform}\left( a, b \right)$ distributions, where $b-a=0.2$ ($u_{0}$: $a=0$, $b=0.2$; $v_{0}$ and $r_{0}$: $a=0.1$, $b=0.3$; $c_{0}$: $a=0.3$, $b=0.5$).


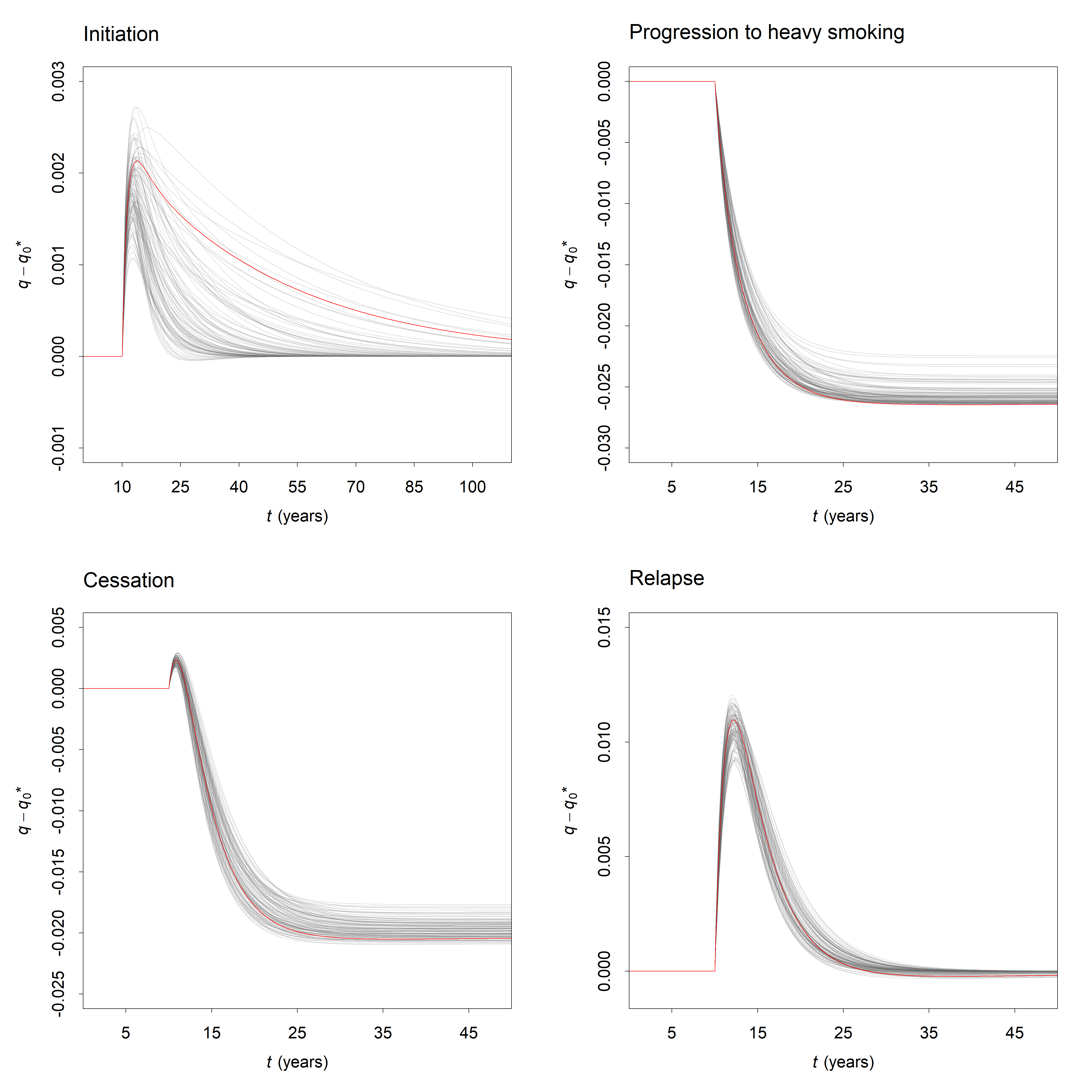


Figure S3. Effects of sustained 10% decreases in per capita rates of initiation, progression to heavily dependent smoking, and relapse ($u$, $v$, and $r$, respectively) and a sustained 10% increase in the per capita cessation rate for less dependent smokers ($c$) on the proportion of heavily dependent smokers in the total smoking population (expressed as the deviation from the equilibrium proportion obtaining prior to the rate change, i.e., $q_{0}^{*}$). The grey lines are derived from the sensitivity analyses; results obtained assuming the parameter values in Table 1 of the paper are shown in red. All rate changes occur at time $t=10$.

Supplementary appendix 3

Markov chain Monte Carlo (MCMC) simulation methods

Posterior simulation was performed using Stan ver. 2.19.2 (http://mc-stan.org/; Carpenter et al., 2017). Normal prior distributions with mean 0 and standard deviation 0.5 were specified for the fractional increase rates $\delta_{u}$, $\delta_{v}$, $\delta_{c}$, and $\delta_{r}$, the initial values for the per capita rates $u$, $v$, and $r$, and the time series-specific standard deviations $\sigma_{i}$ (the prior distributions for the initial per capita rates and the standard deviations were truncated at 0, constraining these parameters to be positive). Priors for the remaining parameters were derived from National Drug Strategy Household Survey (NDSHS) data (Australian Institute of Health and Welfare, 2017; see Table S1). For the initial numbers of current and former smokers and the initial cessation rate (i.e., the total number of smokers quitting for 1 month or more per year), we specified lognormal priors with modes equal to the 2001 NDSHS estimates and standard deviations equal to 5% of the mode. NDSHS data on numbers of pack-a-day smokers (those smoking ≥ 20 cigarettes per day) were available for 2010‒2016 only, so the initial proportion of pack-a-day smokers in the smoking population, $q_{0}$, was estimated using a simple linear regression model predicting $q$ from NDSHS estimates of mean weekly cigarette consumption. We specified a normal prior for $q_{0}$ with mean equal to the predicted value for 2001 and standard deviation equal to 25% of the mean ($q_{0}$ was constrained to lie between 0 and 1 by truncating the prior distribution). Note that the priors specified for the initial number of current smokers ($L_{0}+H_{0}$), the initial total cessation rate ($c_{0}L_{0}+\lambda c_{0}L_{0}$), and $q_{0}$ imply an informative prior distribution for the initial per capita cessation rate for less dependent smokers, $c_{0}$ (see Figure S5).

Four Markov chains, each initialised with parameter values sampled from the joint prior distribution, were run in parallel for 4000 iterations; we used the final 2000 iterations from each chain (8000 samples in total) for posterior inference (i.e., the initial half of each chain was discarded as warmup; see Gelman et al., 2014). Potential scale reduction factors ($\hat{R}$) calculated for all parameters were less than 1.01, indicating approximate convergence to the posterior distribution (see Gelman et al., 2014, pp. 281‒286). Trace plots for selected parameters are presented in Figure S4. Effective sample sizes ($\hat{n}_{eff}$; Gelman et al., 2014) were greater than 10^3^ for all parameters.

Table S1. System dynamics model parameter values and prior distributions assumed in the Markov chain Monte Carlo (MCMC) analysis. (Prior distributions are given using Stan notation.)

| Parameter |  | Symbol(s) | Value or prior | Reference(s) |
| --- | --- | --- | --- | --- |
|  |  |  |  |  |
| Initial population |  | *P*_0_ | 15465971 | Australian Bureau of Statistics (2020) |
| Initial fractional population growth rate |  | *g*_0_ | 0.01748 |  |
| Fractional rate of decrease in the population growth rate |  | *δ_g_* | 0.005349 |  |
| Initial number of current smokers |  | *L*_0_ *+ H_0_* | lognormal(15.1030, 0.0498) |  |
| Initial proportion of heavily dependent smokers in the smoking population |  | *q*_0_ | normal(0.4322, 0.1080) T[0, 1] |  |
| Initial number of smokers quitting per year |  | *c*_0_*L*_0_ *+ λ*c_0_*H_0_* | lognormal(13.7087, 0.0498) |  |
| Initial number of former smokers |  | *F*_0_ | lognormal(15.2246, 0.0498) |  |
| Per capita mortality rate for less dependent smokers |  | *μ* | 0.01197 | Australian Bureau of Statistics (2017), Banks et al. (2015) |
| Mortality rate ratio for heavily dependent smokers |  | *ζ* | 1.6194 | Banks et al. (2015) |
| Mortality rate ratio for former smokers |  | *η* | 0.6712 | Banks et al. (2015) |
| Cessation rate ratio for heavily dependent smokers |  | *λ* | 0.3753 | Australian Institute of Health and Welfare (2017) |
| Initial per capita initiation rate |  | *u*_0_ | normal(0, 0.5) T[0, ] |  |
| Initial per capita rate of progression to heavily dependent smoking |  | *v*_0_ | normal(0, 0.5) T[0, ] |  |
| Initial per capita relapse rate |  | *r*_0_ | normal(0, 0.5) T[0, ] |  |
| Fractional rate of increase in the per capita initiation rate |  | *δ_u_* | normal(0, 0.5) |  |
| Fractional rate of increase in the per capita rate of progression to heavily dependent smoking |  | *δ_v_* | normal(0, 0.5) |  |
| Fractional rate of increase in the per capita cessation rate for less dependent smokers |  | *δ_c_* | normal(0, 0.5) |  |
| Fractional rate of increase in the per capita relapse rate |  | *δ_r_* | normal(0, 0.5) |  |
|  |  |  |  |  |


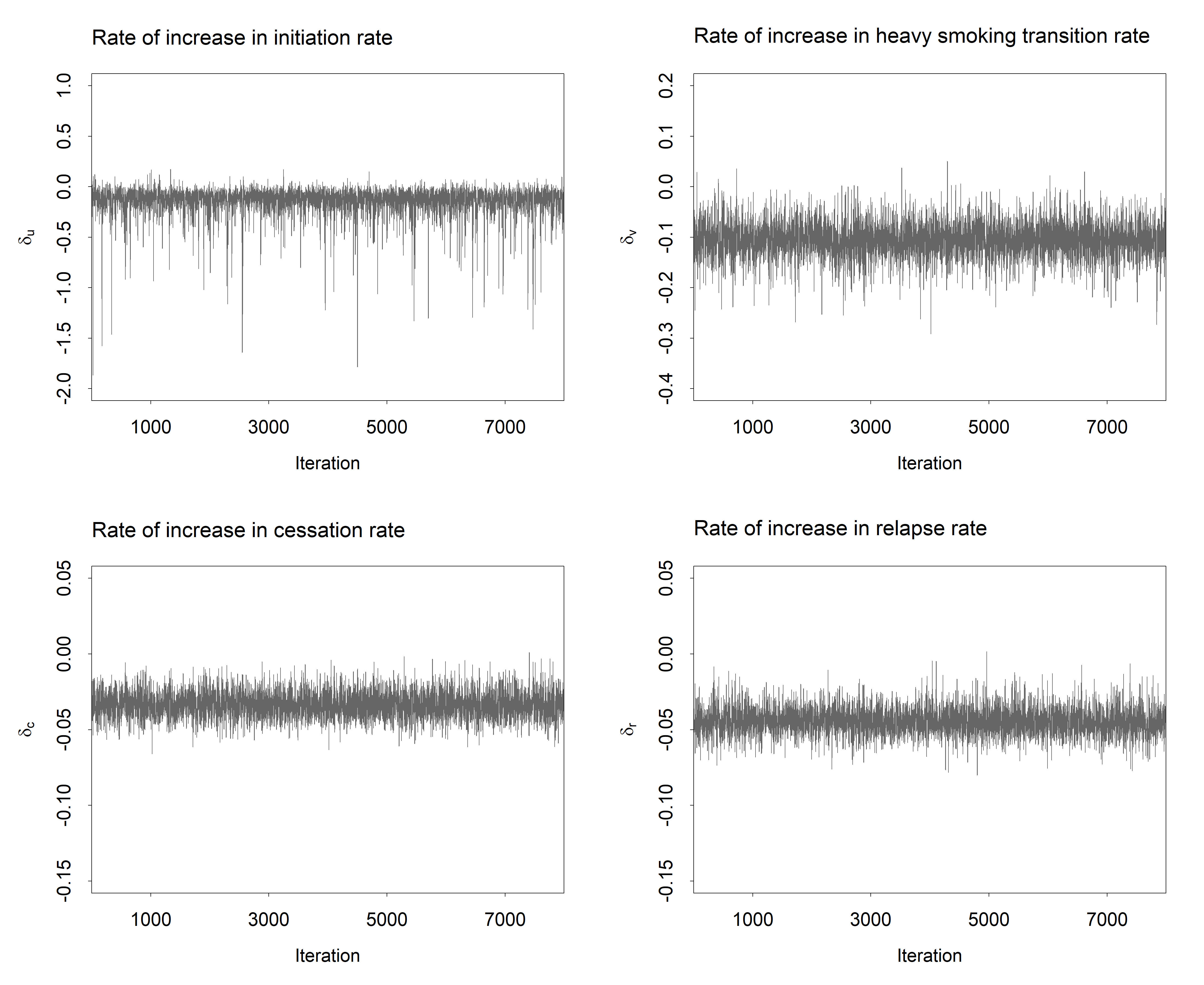


Figure S4. Trace plots for the fractional rates of increase in per capita rates of initiation, progression to heavily dependent smoking, cessation, and relapse ($\delta_{u}$, $\delta_{v}$, $\delta_{c}$, and $\delta_{r}$). Post-warmup samples from the four separate Markov chains are concatenated in each plot, i.e., samples 1‒2000 are from the first chain, samples 2001‒4000 are from the second chain, and so on.

References

Australian Bureau of Statistics, 2017. Deaths, Australia, 2016. Cat. no. 3302.0. Australian Bureau of Statistics, Canberra.

Australian Bureau of Statistics, 2020. Australian demographic statistics, Sep 2019. Cat. no. 3101.0. Australian Bureau of Statistics, Canberra.

Australian Institute of Health and Welfare, 2017. National Drug Strategy Household Survey 2016: detailed findings. Drug Statistics series no. 31. Cat. No. PHE 214. Australian Institute of Health and Welfare, Canberra.

Banks, E., Joshy, G., Weber, M. F., Liu, B., Grenfell, R., Egger, S., Paige, E., Lopez, A. D., Sitas, F., Beral, V., 2015. Tobacco smoking and all-cause mortality in a large Australian cohort study: findings from a mature epidemic with current low smoking prevalence. BMC Med. 13, 38.

Carpenter, B., Gelman, A., Hoffman, M. D., Lee, D., Goodrich, B., Betancourt, M., Brubaker, M. A., Guo, J., Li, P., Riddell, A., 2017. Stan: a probabilistic programming language. J. Stat. Softw. 76 (1), 1‒32.

Gelman, A., Carlin, J. B., Stern, H. S., Dunson, D. B., Vehtari, A., Rubin, D. B., 2014. Bayesian data analysis. CRC Press, Boca Raton.

Supplementary appendix 4

Derivation of the equilibrium proportion of heavily dependent smokers in a smoking population ($q$)

At equilibrium, numbers of less dependent, heavily dependent, and former smokers ($L$, $H$, and $F$, respectively) are constant, i.e., their rates of change are equal to 0, so we have the equilibrium conditions:

$${dL}/{dt}=u\left( P-L^{*}-H^{*}-F^{*} \right)+rF^{*}-vL^{*}-cL^{*}-\mu L^{*}=0$$

$${dH}/{dt}=vL^{*}-\lambda cH^{*}-\zeta\mu H^{*}=0$$

$${dF}/{dt}=cL^{*}+\lambda cH^{*}-rF^{*}-\eta\mu F^{*}=0$$

where $t$ is time in years, $L^{*}$, $H^{*}$, and $F^{*}$ are the equilibrium numbers of less dependent, heavily dependent, and former smokers, respectively, and all other notation is the same as in Figure 1 of the paper. Note that the population ($P$) and per capita rates of smoking behaviour change (i.e., $u$, $v$, $c$, and $r$) are assumed to be constant (consistent with the simulations presented in the paper). The equilibrium proportion of heavily dependent smokers in the total smoking population can be calculated as $q^{*}={H^{*}}/\left( L^{*}+H^{*} \right)$, where $L^{*}$ and $H^{*}$ are derived from the equilibrium conditions above.

Solving the second equilibrium condition (i.e., $vL^{*}-\lambda cH^{*}-\zeta\mu H^{*}=0$) for $H^{*}$, and substituting into the equation for $q^{*}$ yields:

$$q^{*}=\frac{{vL^{*}}/\left( \lambda c+\zeta\mu\right)}{L^{*}+{vL^{*}}/\left( \lambda c+\zeta\mu\right)}=\frac{v}{v+\lambda c+\zeta\mu}$$

so, the equilibrium proportion of heavily dependent smokers in the total smoking population depends only on the per capita rate of progression to heavily dependent smoking, $v$, and the per capita cessation and mortality rates for heavily dependent smokers ($\lambda c$ and $\zeta\mu$, respectively).

To see why an increase in the per capita cessation rate for less dependent smokers, $c$, results in a decrease in the equilibrium proportion of smokers who are heavily dependent, note that as the number of less dependent smokers declines (due to the increase in $c$), the number of less dependent smokers progressing to heavy smoking per year also declines (since the rate of progression to heavily dependent smoking is equal to the number of less dependent smokers multiplied by the per capita rate $v$). At the same time, the per capita cessation rate for heavily dependent smokers, $\lambda c$, increases (because $c$ increases), actively reducing the number of heavy smokers in the smoking population. Combined, the decline in the number of less dependent smokers progressing to heavily dependent smoking per year and the increase in the heavy smoker cessation rate result in a proportional decrease in the number of heavily dependent smokers that is greater than the proportional decrease in the number of less dependent smokers due to the increase in $c$. Denoting the equilibrium numbers of less dependent smokers prior to and after an increase in the per capita cessation rate from $c$ to $\gamma c$ as $L_{0}^{*}$ and $L_{1}^{*}$, respectively, the equilibrium numbers of heavily dependent smokers before and after the rate increase are, respectively, $H_{0}^{*}={vL_{0}^{*}}/\left( \lambda c+\zeta\mu\right)$ and $H_{1}^{*}={vL_{1}^{*}}/\left( \lambda\gamma c+\zeta\mu\right)$ (these equations are derived from the second equilibrium condition above). The proportional decrease in the equilibrium number of heavily dependent smokers resulting from the increase in the per capita cession rate $c$ is therefore:

$$\frac{-\left( H_{1}^{*}-H_{0}^{*} \right)}{H_{0}^{*}}=\frac{-\left[ {vL_{1}^{*}}/\left( \lambda\gamma c+\zeta\mu\right)-{vL_{0}^{*}}/\left( \lambda c+\zeta\mu\right) \right]}{{vL_{0}^{*}}/\left( \lambda c+\zeta\mu\right)}=1-\frac{L_{1}^{*}}{L_{0}^{*}}\cdot\frac{\lambda c+\zeta\mu}{\lambda\gamma c+\zeta\mu}$$

Because $\gamma$ is greater than 1 (the per capita cessation rate increases, so $\gamma c$ > $c$), $\lambda\gamma c+\zeta\mu$ exceeds $\lambda c+\zeta\mu$, and $\left( \lambda c+\zeta\mu\right)/\left( \lambda\gamma c+\zeta\mu\right)<1$. Thus, $\left( {L_{1}^{*}}/{L_{0}^{*}} \right)\left( \lambda c+\zeta\mu\right)/\left( \lambda\gamma c+\zeta\mu\right)<{L_{1}^{*}}/{L_{0}^{*}}$, and ${-\left( H_{1}^{*}-H_{0}^{*} \right)}/{H_{0}^{*}}$ is greater than the proportional decrease in the number of less dependent smokers, equal to ${-\left( L_{1}^{*}-L_{0}^{*} \right)}/{L_{0}^{*}}=1-{L_{1}^{*}}/{L_{0}^{*}}$.

Supplementary appendix 5

Posterior distributions estimated for the initial value parameters in the system dynamics model


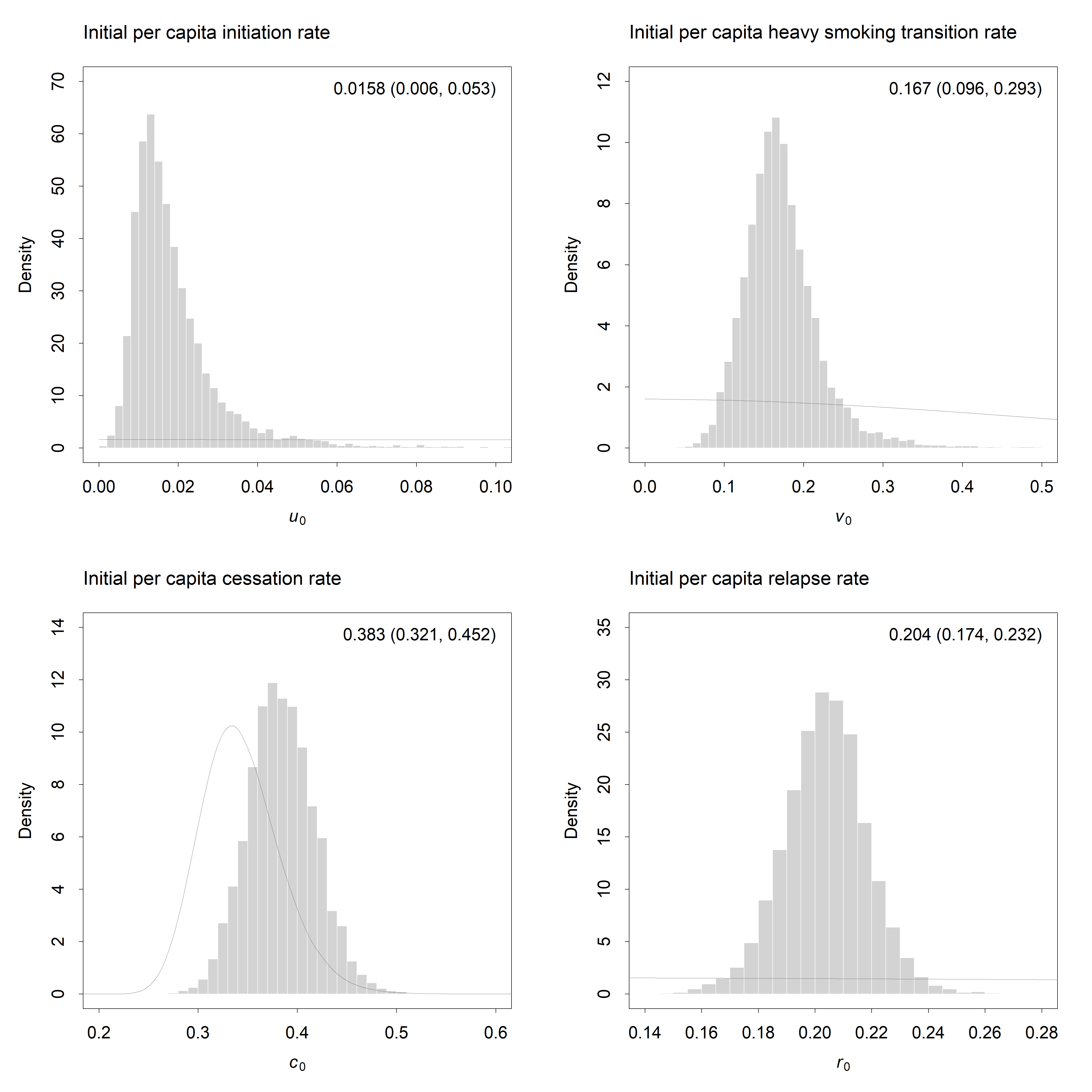


Figure S5. Marginal posterior distributions estimated for initial per capita rates of smoking behaviour change. Median estimates and central 95% posterior intervals are shown in the top right corner. Prior distributions are plotted as smooth curves.


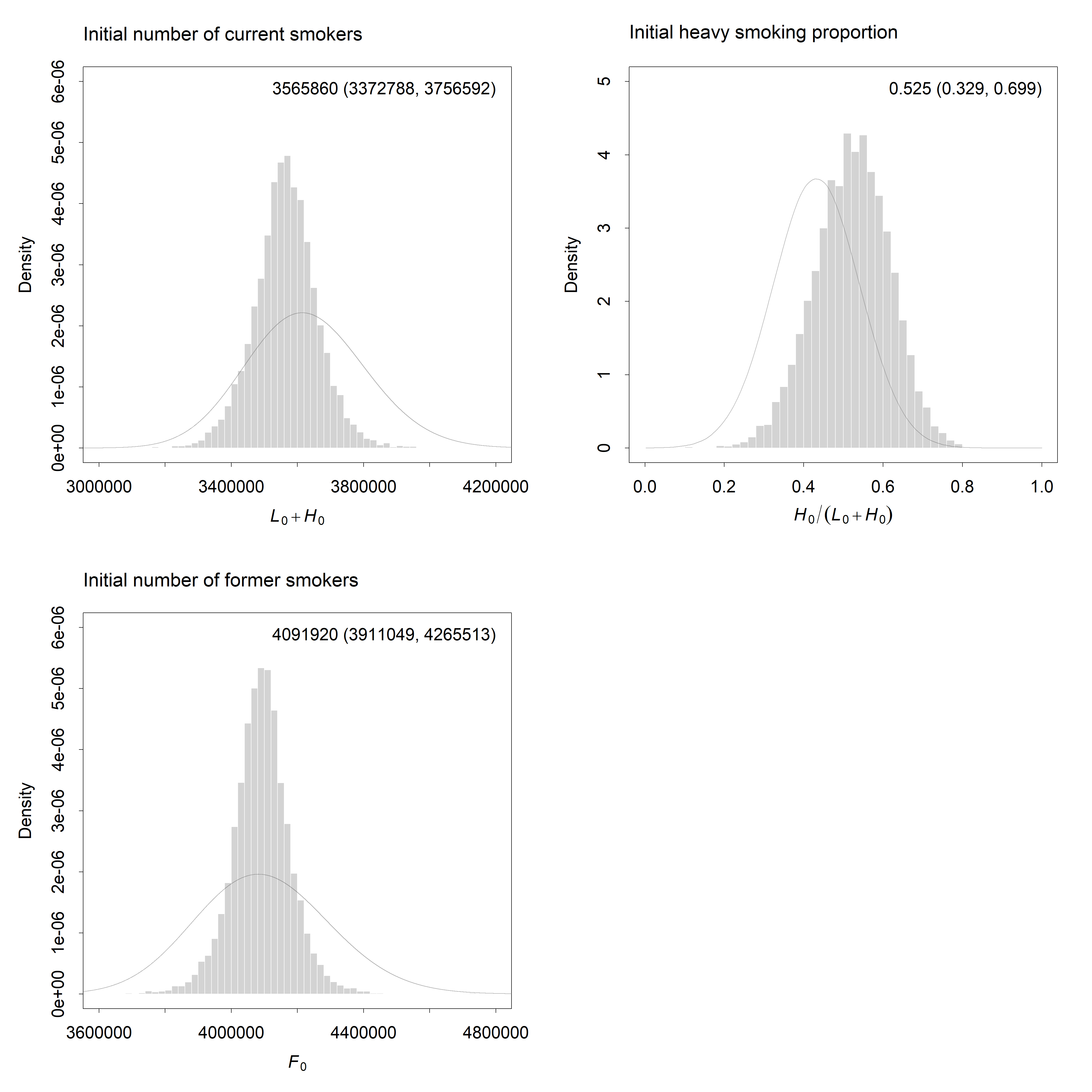


Figure S6. Marginal posterior distributions estimated for initial numbers of current and former smokers and the initial proportion of heavy smokers (i.e., ≥ 20 cigarettes per day) in the Australian smoking population. Median estimates and central 95% posterior intervals are shown in the top right corner. Prior distributions are plotted as smooth curves.
